# Emergency centre reorganization in preparation to the COVID-19 pandemic: A district hospital’s dynamic adaptation response

**DOI:** 10.4102/phcfm.v12i1.2514

**Published:** 2020-09-16

**Authors:** Phillip P. Furstenburg, Suzan N. Mukonkole, Crispin N. Kibamba, Ashley Kuiler, Nondumiso Ngemntu, Sa’ad Lahri, Daniël J. van Hoving, Kitesh Moodley, Elaine Erasmus

**Affiliations:** 1 Department of Emergency Medicine, Faculty of Medicine and Health Sciences, Khayelitsha Hospital, Cape Town, South Africa; 2 Division of Emergency Medicine, Department of Family Medicine and Emergency Medicine, Faculty of Medicine and Health Sciences, Stellenbosch University, Cape Town, South Africa; 3 Department of Critical Care Nursing, Faculty of Medicine and Health Sciences, Western Cape College of Nursing, Cape Town, South Africa

**Keywords:** emergency centre, COVID-19, pandemic, district hospital, Cape Town

## Abstract

The COVID-19 global pandemic forced healthcare facilities to put special isolation measures in place to limit nosocomial transmission. Cohorting is such a measure and refers to placing infected patients (or under investigation) together in a designated area. This report describes the physical reorganisation of the emergency centre at Khayelitsha Hospital, a district level hospital in Cape Town, South Africa in preparation to the COVID-19 pandemic. The preparation included the identification of a person under investigation (PUI) room, converting short stay wards into COVID-19 isolation areas, and relocating the paediatric section to an area outside the emergency centre. Finally, we had to divide the emergency centre into a respiratory and non-respiratory side by utilising part of the hospital’s main reception. We are positive that the preparation and reorganization of the emergency centre will limit nosocomial transmission during the expected COVID-19 surge. Our experience in adapting to COVID-19 may have useful implications for ECs throughout South Africa and in low-and-middle income countries that are preparing for this pandemic.

## Introduction

The COVID-19 pandemic is overstretching healthcare systems across the globe, and various special measures have been put in place to contain and limit the spread of the disease. The role of the emergency centre (EC) as a frontline of defence is pivotal in ensuring that suspected COVID-19 cases are contained and isolated from arrival. This is crucial, given the significance of nosocomial transmission. In line with the national strategy of containment, we are undertaking a dynamic adaptive response with infrastructural modifications to accommodate the surge of patients, improve infection control and prevent nosocomial transmission.

Infected patients (or patients under investigation) should ideally be managed in single rooms, but most ECs have limited isolation capacity. Cohorting refers to placing patients infected with the same pathogens (or under investigation) together in a designated area and requires that ECs re-think the utilisation of floor space.^1^

This report describes the physical reorganisation of the EC at Khayelitsha Hospital, a district-level hospital in Cape Town, South Africa, in preparation for the COVID-19 pandemic. The EC sees around 3000 new patients per month with a reported inpatient bed occupancy level at ± 130%.^2^

Our experience in adapting to COVID-19 may have useful implications for ECs throughout South Africa and in low- and middle-income countries that are preparing for this pandemic.

### Original lay out

**FIGURE 1:**
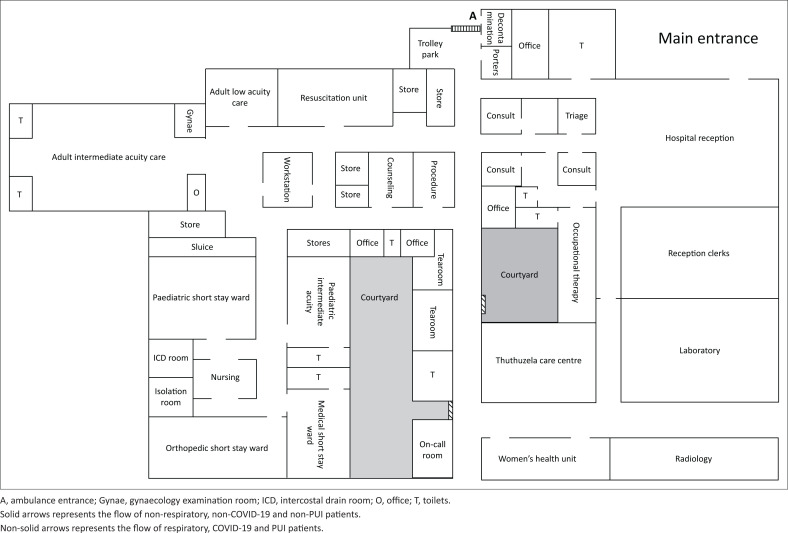
Original layout: Phased reorganization of Khayelitsha Hospital’s emergency centre in preparation to the COVID-19 pandemic.

The EC before the pandemic consisted of a single resuscitation room (four beds, one paediatric radiant incubator), one adult intermediate acuity area (22 trolleys), one paediatric intermediate acuity area (two beds, six chairs), one low adult acuity area (20 chairs), one gynaecology examination room (one bed), a triage area (one triage room, two consultation rooms, two waiting areas), one procedure room, one counselling and/or bereavement room and a short stay ward (areas: paediatric (six cots), adult medical (eight beds), adult orthopaedic (eight beds), one isolation room, one intercostal drain room (eight chairs)) (Figure 1). Mental Health Care patients’ were seen and observed in the adult intermediate acuity area until they could move to the psychiatry ward. The re-organisation was planned to ensure minimal changes, but all the required changes couldn’t happen simultaneously. A phased approach further allowed improvement in flow as additional space were required with time.

### Phase 1

**FIGURE 2:**
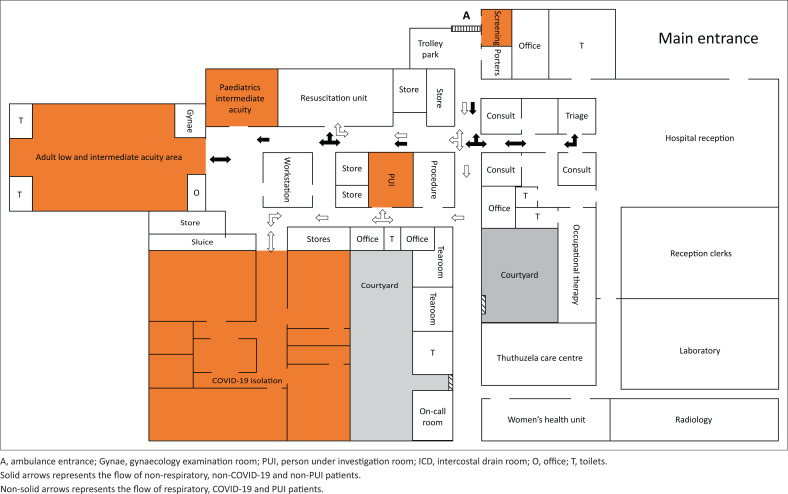
Phase 1: Phased reorganization of Khayelitsha Hospital’s emergency centre in preparation to the COVID-19 pandemic.

The described reconfiguration of the EC extended over a six-week period (Figure 2). The first changes were implemented on 12 March 2020 when the second COVID-19 case was confirmed in Cape Town. It involved erecting a screening station in front of the main hospital entrance and converting the decontamination room into a screening area at the ambulance entrance. The counselling/bereavement room was converted into a person under investigation (PUI) room. A COVID-19 isolation ward was created by relocating the adult short stay wards to the outpatients’ department (which was closed because of the pandemic) and by merging and moving the paediatric intermediate acuity area and paediatric short stay area to the adult low acuity area. Patients who were usually managed within the adult low acuity area were moved into the adult intermediate acuity area.

### Phase 2

**FIGURE 3:**
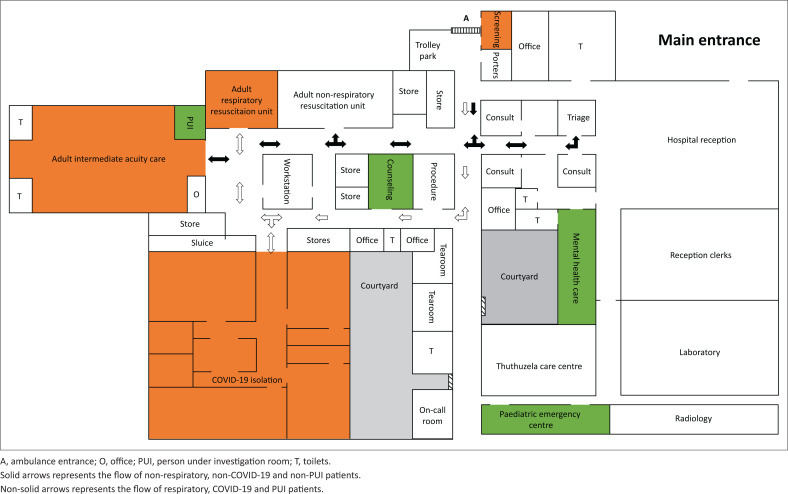
Phase 2: Phased reorganization of Khayelitsha Hospital’s emergency centre in preparation to the COVID-19 pandemic.

The institution of a national lockdown on 26 March 2020 triggered the next phase,^3^ and expanded testing within the hospital was undertaken (Figure 3). Phase 2 involved the complete separation of adult and paediatric patients, by moving the paediatric intermediate acuity area out of the main EC and into an area previously used for Women’s Health. The gynaecology examination room became the new PUI room and the phase 1 paediatric intermediate area (formerly the adult low acuity area) became the respiratory resuscitation area. Mental Healthcare patients were moved from the original adult intermediate acuity area to the room previously used by occupational therapy.

### Phase 3

**FIGURE 4:**
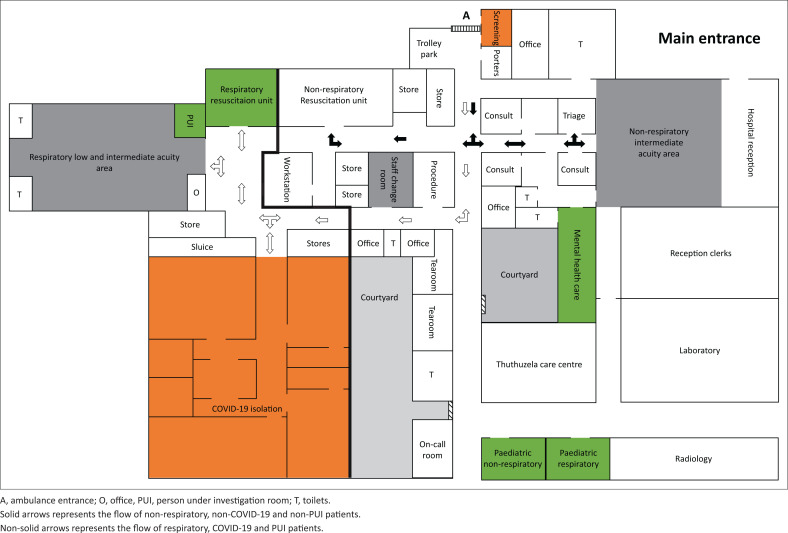
Phase 3: Phased reorganization of Khayelitsha Hospital’s emergency centre in preparation to the COVID-19 pandemic.

The EC was eventually divided into a respiratory and non-respiratory side (Figure 4). Presently, the respiratory side consists of a respiratory resuscitation room (the original adult low acuity area), a PUI room (formerly the gynaecology examination room), and the adult low and intermediate acuity area. The availability of wall oxygen points mainly determined which side was converted into the respiratory side. The non-respiratory area includes the original resuscitation area, procedure room, triage area and a 14-bed adult intermediate acuity area. The latter was created by cordoning off about two-thirds of the hospital’s original main reception area (it has a steel roller door, initially used to create a safe overnight waiting area for EC patients that were≈discharged at night). The original counselling/bereavement room is now being used as the staff’s change room for donning and doffing personal protective gear. The relocated paediatric EC was also divided into a respiratory side (with a resuscitation room, a triage room, PUI room and admission area) and a non-respiratory side (with a resuscitation room, triage and assessment room and short stay area).

## Additional staffing and equipment requirements

Two doctors and four nurses were added to each shift. All the nurses and one doctor were acquired as locums, while the second doctor was relocated from other departments. All additional equipments (resuscitation, procedure and medication trolleys) were redistributed from other areas, e.g. out patients department and skills laboratory.

## Limitations

Our strategy is based on isolating and cohorting patients with respiratory symptoms, and the current case definition may not be suitable in the later stages of this pandemic due to the protean clinical manifestations of COVID-19.^4^ Ideally, three completely separated areas (with separate entrances) are needed to host confirmed cases, patients under investigation and confirmed negative patients, but the physical layout and limited resources (e.g. duplication of staff and equipment) prevents this.

## Future direction

Further reorganization plans include construction of a physical barrier between the respiratory and non-respiratory side as well as perspex partitioning in the respiratory area to create physical barriers between the patients. A dedicated respiratory triage room and the creation of a tearoom for exclusive use by staff members on the respiratory side is also planned. Additional escalation plans are being put in place by the Western Cape Department of Health, including dedicated field hospitals to host patients with low to intermediate acuity (both confirmed and under investigation).

## Conclusion

The pandemic is rapidly evolving and we are currently experiencing community spread. Dynamic reassessment of our workflow and patient-streaming pathways are being done daily. Restructuring and reorganising of the EC requires multidisciplinary involvement of healthcare providers and hospital managers; all working towards a common goal of staff and patient safety. We are cautiously optimistic that the preparation and reorganization of the EC will limit nosocomial transmission during the expected COVID-19 surge; however, only time will tell.
